# The potential of the amaranth collection maintained at VIR in the context of global plant breeding and utilization trends

**DOI:** 10.18699/vjgb-24-81

**Published:** 2024-11

**Authors:** D.V. Sokolova, A.E. Solovieva, A.M. Zaretsky, T.V. Shelenga

**Affiliations:** Federal Research Center the N.I. Vavilov All-Russian Institute of Plant Genetic Resources (VIR), St. Petersburg, Russia; Federal Research Center the N.I. Vavilov All-Russian Institute of Plant Genetic Resources (VIR), St. Petersburg, Russia; Federal Research Center the N.I. Vavilov All-Russian Institute of Plant Genetic Resources (VIR), St. Petersburg, Russia; Federal Research Center the N.I. Vavilov All-Russian Institute of Plant Genetic Resources (VIR), St. Petersburg, Russia

**Keywords:** Amaranthus L., valuable traits, breeding trends, species diversity, VIR’s amaranth collection, Amaranthus L., ценные признаки, направления селекции, видовое разнообразие, коллекция амаранта ВИР

## Abstract

Amaranth is an ancient crop of the family Amaranthaceae, but it is fairly new to Russia. Its seeds and leaf biomass contain a high-quality gluten-free protein, fatty acids, squalene (a polyunsaturated hydrocarbon), flavonoids, vitamins, and minerals. A comprehensive study of amaranth, enhancement of its breeding, and development of new cultivars will contribute to food quality improvement through the use of plant raw materials enriched for wholesome and highly nutritious components. At present, selection and hybridization still remain the main amaranth breeding techniques. Meanwhile, mutation breeding and polyploidy have been successfully employed to increase its seed yield and protein content. The genes encoding amaranth proteins have been used to produce transgenic plants of potato, bread wheat, and maize. Despite the great potential of amaranth, little research has been dedicated to the study of its genomics, concentrating mainly on the identification of its species diversity. Targets of breeding practice for amaranth include such characteristics as large size and nonshattering of seeds, short stem, earliness, high yield, cold hardiness, synchronized maturation, resistance to pests and diseases, and high nutritional value, including the content and quality of protein, lipids, squalene, and bioactive compounds. A unique collection of amaranth maintained at the N.I. Vavilov All-Russian Institute of Plant Genetic Resources (VIR) currently incorporates 570 accessions from various countries. For 70 years it has been replenished with local varieties, commercial cultivars, and wild species supplied by collecting missions, research centers, botanical gardens, genebanks, and experimental breeding stations from all over the world. Long-standing studies have resulted in the formation of trait-specific groups of accessions, with high yields of seeds and leaf biomass, earliness, cold hardiness, high protein content in seeds and biomass, short stems, and resistance to seed shattering, earmarked for vegetable or ornamental purposes. The gene pool of amaranth preserved at VIR can provide unlimited opportunities for breeding and meet the needs of the country’s population, enriching the human diet with ingredients produced from such a health-friendly and useful crop.

## Abstract (ru)

Амарант – древняя культура семейства Амарантовые (Amaranthaceae). Для России это достаточно новая сельскохозяйственная культура. В семенах и листовой биомассе содержатся высококачественный безглютеновый белок, жирные кислоты, полиненасыщенный углеводород сквален, флавоноиды, витамины и минералы. Комплексное изучение амаранта, развитие его селекции и создание новых сортов являются крайне важным направлением для решения проблемы повышения качества пищевой продукции путем использования растительного сырья, обогащенного полезными и высокопитательными компонентами. На сегодняшний день основными методами селекционной работы с амарантом остаются отбор и гибридизация. Методы мутационной селекции и полиплоидии были успешно использованы для увеличения урожайности семян и содержания белка. С помощью генов, кодирующих белки амаранта, созданы трансгенные растения картофеля, мягкой пшеницы и кукурузы. Несмотря на большой потенциал амаранта, изучению его геномики посвящено не много исследований, направленных главным образом на идентификацию видового разнообразия. В направления селекционной работы с амарантом входят такие признаки, как «крупность и неосыпаемость семян», «низкорослость», «скороспелость», «высокая урожайность», «холодостойкость», «синхронность созревания», «устойчивость к вредите лям и болезням», «высокая питательная ценность»: содержание и качество белка, липидов, сквалена, биологически активных соединений. Уникальная коллекция амаранта Всероссийского института генетических ресурсов растений им. Н.И. Вавилова (ВИР) включает 570 образцов из различных стран мира. На протяжении 70 лет она пополнялась местными, селекционными сортами и дикими видами за счет экспедиций, поступлений из научноисследовательских институтов, ботанических садов, генбанков и опытных селекционных станций всего мира. В результате многолетнего изучения были сформированы признаковые группы образцов с высокой урожайностью семян и листовой биомассы, скороспелые и холодостойкие, с повышенным содержанием белка в семенах и биомассе, низкорослые, устойчивые к осыпанию семян, овощного и декоративного направления использования. Сохраняемый в ВИР генофонд амаранта способен предоставлять неограниченные возможности для се лекции и восполнять нужды населения страны, обогащая питательный рацион продуктами из этой здоровой и полезной культуры

## Introduction

The industrialization of agricultural production and the consolidation of the integrated global market ensured sustainable growth of worldwide food supplies induced by higher crop yields. Meanwhile, the innovation processes in agriculture, based on the release of high-yielding crop cultivars and the development of agricultural technologies, dealt with only some of the staple crops (soybean, wheat, rice, maize, sunflower, etc.). The resulting depletion of agricultural biodiversity and gradual replacement of minor crops posed a potential threat to global food security (Khoury et al., 2014; Dawson et al., 2019). The abovementioned crops provide enough calories, but they are deficient in essential amino acids, minerals, and vitamins for maintaining a wholesome and well-balanced human diet, leading to the “hidden” malnutrition of more than two billion people worldwide, whose daily diet consists almost entirely of these crops (Cheng et al., 2015).

Throughout its existence, humankind has used around 3,000 plant species, but only about 150 of them are cultivated commercially (Mangelsdorf, 1966). Other sources report that about 30,000 plant species in the world are edible, but only 7,000 of them are used for food (Ramdwar et al., 2017). Integration of a wide range of “forgotten crops” to improve dietary diversity can improve the quality of human nutrition in many countries and ensure their food security (Mayes et al., 2011; Ebert, 2014; Joshi D.C. et al., 2018).

Amaranth is one of the plants with the potential to become an alternative grain crop on the global scale (Das, 2016). The objective of this study was to make a historical review of amaranth breeding, characterize the genetic diversity of amaranth preserved in the VIR collection, and contemplate its prospects for domestic breeding practice.

Amaranth is an ancient crop, described in botany as gen. Amaranthus L., subfam. Amaranthoideae, fam. Amaranthaceae, ord. Caryophyllales. The genus Amaranthus L. includes, according to various sources, from 60 to 87 species, being one of the top ten taxonomically most complex crops. Along with buckwheat and quinoa, it represents a small group of “pseudocereals” (Saunders, Becker, 1984; Teutonico, Knorr, 1985).

Most of its species are wild or weedy. The grain species of amaranth are Amaranthus cruentus and A. hypochondriacus from Central and North Americas, and A. caudatus of South American origin (Covas, 1994). The history of amaranth cultivation in these regions dates back 5,000 to 7,000 years. According to archaeological records describing the materials collected in the northwest of Argentina, the age of the discovered seeds was dated to the beginning of the Middle Holocene (5,500 to 6,000 BC). The oldest finds of amaranth were registered in a cave in the highland area of Peñas de la Cruz, Department of Antofagasta de la Sierra (3,665 MASL). These data indicated a much earlier use of amaranth – 10,000 and 7,000 BC (Arreguez et al., 2013).

Amaranth was also a very important food source for the population of pre-Hispanic South America (Chagaray, 2005). From ancient times, the Aztec and Mayan tribes used it as a grain crop, second in importance only to maize and legumes (Sauer, 1967; Smith M.E., 1996). Amaranth leaves were also consumed. Mixing whole or ground amaranth grain to prepare bread, porridges, and cakes was quite popular, as well as its ceremonial use in temples. The Aztecs made small figurines of gods from amaranth dough and ate them as part of their rituals (De Montellano, 1990). Most likely, it was the reason why Spanish conquerors strictly prohibited amaranth consumption and cultivation in the early 16th century, which led to its falling into obsolescence for many years (Saunders, Becker, 1984).

The revival of interest in amaranth in the late 20th century was associated with the efforts to study its unique biochemical characteristics, multipurpose utilization prospects, and the C4 photosynthesis mechanism typical of amaranth (Venskutonis, Kraujalis, 2013; Magomedov, Chirkova, 2015). For Russian agriculture it is a fairly new crop, with its enormous potential for growth intensity, productivity, and high complete protein content in seeds and leaf biomass (Kononkov et al., 1999). Therefore, a comprehensive study of amaranth, its improvement through breeding, and development of new cultivars will contribute a great deal to the task of raising the quality of human nutrition by means of employing plant materials enriched with useful and highly nutritious components.

Classification of the genus Amaranthus is hampered by the absence of species-specific and qualitative identifying characters, a wide range of phenotypic variability between species, and introgression and hybridization between weedy and cultivated species (Sauer, 1967; Hauptli, Jain 1978). Many researchers have applied morphological, biochemical, molecular and cytogenetic methods to assess the level of interspecies phylogenetic relationships (Murray, 1940; Costea et al., 2001; Das, 2012; Akin-Idowu et al., 2016). Most of the authors agree that all grain amaranths descended from their weedy progenitor A. hybridus.

The cultivated grain species have close relationships among themselves, but A. hypochondriacus (2n = 32) and A. caudatus (2n = 32) are more closely related to each other than to A. cruentus (2n = 34). A specific feature of A. cruentus is the presence of one copy of chromosome 2, resulting in a haploid number of n = 17 (Singh et al., 2023).

A majority of the Amaranthus representatives are annual herbaceous plants with claret-colored or yellow-green leaves and inflorescences. The anatomical and morphological diversity of amaranth plants pertains to species specificity and growing conditions. Plant height varies from 40 cm to 5 m. The stem is usually erect, striated, and prolifically leafy. Plants of some species exhibit a sprawling semi-accumbent shape. The degree of branching in amaranth plants can be weak, medium, or strong. The plant habit is formed by a combination of features: the position and branching of the main stem, its size, and the shape of the inflorescence. The leaves are stipule-free, alternate or opposite, differing in leaf and margin shapes. The average number of leaves on a plant can reach 250, with the leaf surface area being ca. 7,500–8,000 cm2. The inflorescence is a compound panicle of varying shape, density, and color. The flowers are small, actinomorphic, dioecious, less often bisexual, clustered in the leaf axils. The androecium consists of 5 stamens; the gynoecium, of 3, less often 4 carpels. The superior ovary is unilocular (Das, 2016).

Amaranth is recognized as a “superfood” because of its nutraceutical value, i. e., the content of high-quality gluten-free protein, unsaturated fatty acids, dietary fibers, flavonoids, vitamins (thiamine, riboflavin, ascorbic acid, and niacin), and minerals (calcium, magnesium, and copper, plus sodium, iron, phosphorus, and zinc) (Kononkov et al., 1999; Grobelnik-Mlakar et al., 2009; Palombini et al., 2013; Joshi D.C. et al., 2018; Soriano Garcıa et al., 2018; Sokolova et al., 2021). Its seeds contain methionine (15.8 mg/g total protein) and lysine (55.8 mg/g total protein), ensuring the crop’s higher nutritional value compared to most cereals (Tang, Tsao, 2017).

The amount of lipids in amaranth seeds varies greatly across the species and genotypes, ranging within 1.9–9.7 %. Palmitic, oleic, linoleic and linolenic fatty acids are present in high amounts, accounting aggregately for over 90 % of the total fatty acid content. Amaranth seed oil has proved its therapeutic effect.

The fatty acid composition of amaranth oil is almost similar to that of cereals, but there is a difference: it contains relatively high levels of squalene (C30H50), a polyunsaturated hydrocarbon (Bressani, 1994). Squalene has a wide range of applications in medicine – as an adjuvant in vaccines, or an immunomodulator and antioxidant in complex therapy against a number of diseases, such as diabetes and coronary heart disease – and is also used in cosmetics (Gonor et al., 2006; Huang et al., 2009). There is convincing evidence that squalene reduces the risk of cancer development and controls cholesterol levels in the human organism (Miettinen, Vanhanen, 1994; Rao et al., 1998; Smith T.J., 2000). The ever increasing interest in this compound is explained by its combined therapeutic effect: antioxidant, hypolipidemic, antitoxic, and antidiabetic (Magomedov et al., 2017).

## Uses of amaranth

**Fig. 1.**
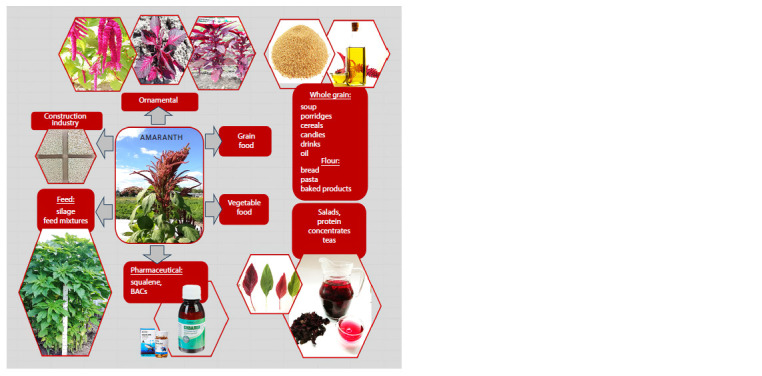
The uses of amaranth (created by the authors).

Cultivated amaranth species are divided into two main groups according to the ways of their utilization: food (vegetable and grain products), and feed. Besides, there are uses less known to the public: ornamental, pharmaceutical, and construction material production (Fig. 1). Such division is arbitrary enough: one and the same cultivar can be used as both feed and food (grain), while the leaves of younger plants belonging to all cultivated species can be consumed fresh as salad ingredients (Ruth et al., 2021; Sokolova et al., 2021).

Initially, amaranth was cultivated for its edible seeds (Central and South America, and mountainous areas in Asia) and as a green vegetable crop (Africa, South Asia, and Southeast Asia). Vegetable amaranth species are widely used for food in India, in the countries of Asia and Southeast Asia, and in Africa, but they are little known in North and South Americas.

Leaves, shoots, and tender juicy stems of vegetable amaranths are used to prepare sauces, soups, or vegetable stews. Young leaves of grain amaranth are also consumed as leafy vegetables. The claret-colored leaves of A. cruentus serve as raw material for the production of teas enriched with amaranth. Amaranth seeds are digestible both in their whole (porridges, cereals, and candies) and milled form (bread, pasta, or baked products) (Das, 2016).

Oil is extracted mainly from the seeds of two amaranth species: A. cruentus and A. hypocondriacus; its content varies within 4.8–8.1 % (He, Corke, 2003; Gamel et al., 2007). The yield of amaranth oil exceeds that of most cereals, but is inferior to oil crops (Ayorinde, 1989; Leon-Camacho et al., 2001). H.P. He and H. Corke (2003) studied the oil content in 104 samples of 30 amaranth species: this indicator showed significant variability, depending on the genotype, growing environments, and the effect of abiotic factors. Moreover, wild forms of amaranth matched its cultivars in their oil content levels, thus confirming their value for breeders (Table 1).

**Table 1.**
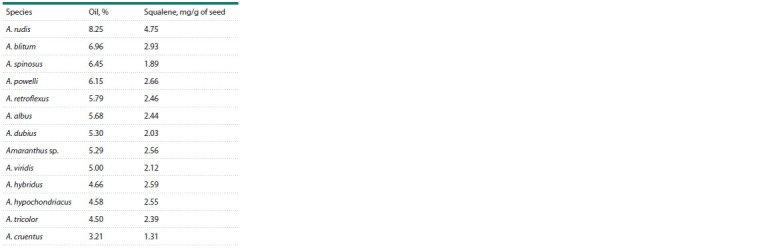
The content of oil and squalene in the seeds of different amaranth species, according to (He, Corke, 2003)

The use of amaranth for animal feed implies utilization of the aboveground plant biomass. However, protein-rich amaranth seeds are also included in feed mixtures. The yield of green biomass averages 85–103 t/ha, with the dry matter yield of 15.7–16.7 t/ha (Abbasi et al., 2012; Shadi et al., 2020). The biomass yield of A. hypochondriacus cultivars in the northern regions of China can reach 130 t/ha, with the dry matter yield of 20 t/ha (Sun G.Q. et al., 2017). H. Shadi et al. (2020) reported a higher level of nondegradable digestible protein in amaranth silage than in the one made from maize, which attests to its higher efficiency. High crude protein and low lignin content, low nitrate and oxalic acid levels account for the high potential of amaranth silage as a feed for ruminants (Sleugh et al., 2001; Rezaei et al., 2009). However, amaranth leaf biomass contains antinutrients, such as trypsin inhibitors, saponins, alkaloids, and oxalates – the feature that reduces the crop’s nutritional value and requires appropriate improvement through breeding (Cheeke et al., 1981).

Many amaranths demonstrate noticeable ornamental properties (Sauer, 1967). A new trend in amaranth utilization is the use of its lignified leading shoots in the construction business to manufacture various wood-based panels (Evon et al., 2021).

Amaranth cultivation technologies today are quite different from the agricultural practices of early civilizations, when neither machinery nor advanced techniques were available. Modern agricultural mechanization methods make it possible to achieve higher yields and profits.

## Amaranth improvement through breeding

Cultivated amaranth is characterized by its rich genetic diversity and environmental plasticity. It is predominantly an autogamous crop, but its percentage of outbreeding is 5–39 %, a level sufficient to ensure gene flow among populations (Hauptli, Jain, 1985). The crop’s diversified mechanism of reproduction is feasible due to the ratio and distribution of male and pistillate flowers in its inflorescences. Obligate allogamous species include A. tuberculatus, A. palmeri, A. arenicola, and A. rudis, all of them dioecious

For amaranth, breeding trends depend on the ways of its utilization. Negative features of this crop, limiting its commercial cultivation and requiring improvement by breeders, include: too small seeds, great plant height, asynchronous seed ripening, seed shattering, size and rigidity of the stem holding a large and heavy inflorescence, and dense structure of the latter (Kauffman, 1984). The great height of grain amaranth plants (1.8–2.5 m) is a negative trait, making its harvesting difficult. Such plant forms are prone to lodging and need to be supported, which leads to an increase in cultivation costs. Plant height variability within amaranth cultivars allows breeders to select short-stemmed plants and use them in crosses

Breeding work with grain amaranths should be targeted mainly at such useful traits as large size and nonshattering of seeds, short stem, earliness, high yield, cold hardiness, synchronized maturation, resistance to pests and diseases, and high nutritional value, including the content and quality of protein, lipids, and bioactive compounds. A desirable target for breeders working with vegetable amaranths is greater bushiness to ensure the possibility of repeated cutting (Sreelathakumary, Peter, 1993).

## Conventional breeding techniques

Selection

This approach was most popular in the United States and India, where selection from local populations resulted in the release of amaranth cultivars still in use today. Germplasm lines from the working collection of the Rodale Research Center, Pennsylvania, USA, became the progenitors of the majority of amaranth cultivars developed in the United States and China (Stallknecht, Schulz-Schaefer, 1993). The first A. cruentus lines registered by the Crop Science Society of America were Montana-3, with white seeds and high yield, and Montana-5, combining the traits of Montana-3 with synchronized ripening (Schulz-Schaeffer et al., 1989a, b). Later, the lodging-resistant cv. Amont was obtained through selection from Montana-3 at Montana State University (Schulz-Schaeffer et al., 1991). Charles S. Kauffman from the Rodale Research Center presented the results of his research and successful selection of grain amaranths for at a number of important traits: seed size, synchronized maturation, increased protein content in seeds, and resistance to shattering and pests. It was also there that K-432, a semi-dwarf line with plant height not exceeding 92 cm, was produced by means of selection among short-stemmed forms of amaranth (Kauffman, 1992).

High-yielding, mid-season, and dwarf forms suitable for mechanized harvesting were selected by screening local grain amaranth accessions from the Mexican plant genetic resources collection (Espitia, 1992). Three well-known high-yielding cultivars of A. caudatus, Oscar Blanco, Noel Vietmeyer, and Alan Garcia, were developed at the University of Cuzco, Peru. Having achieved wide distribution, they are currently cultivated commercially over hundreds of hectares. In Kenya, an improved line of A. hypochondriacus served as a source for the local cultivar Jumla (Kauffman, Weber, 1990; Joshi B.D., Rana, 1991).

An example of effective germplasm utilization in India was the development of the grain amaranth cultivar Annapurna in 1984 at the NBPGR Regional Station in Shimla, India; it was obtained as a pure line of A. hypochondriacus from source material of local origin (Joshi B.D. et al., 1983). The average seed yield of cv. Annapurna is 2.25 t/ha, and the protein content is 15 %. The same station released cv. Durga, resistant to lodging, major diseases and pests, and early-ripening cvs. Gujarat Amaranth-1, Gujarat Amaranth-2, Kapilasa, and Suvarna (Raiger, Bhandari, 2012).

The experiments conducted in 1977–1988 in Minnesota, USA, reported the highest grain yield of 1.72 t/ha for promising cultivars (Myers, Putnam, 1988). Modern cultivars developed by domestic breeders demonstrate the yields of 2.35 t/ha (cv. Karakula) and 2.09 t/ha (cv. Voronezhsky) (State Register for Selection Achievements Admitted for Usage, 2023). It should be taken into account that the presentday agricultural practice for amaranth cultivation is quite different from earlier practices, when neither machinery nor technologies were available. Joint efforts of plant breeders and agricultural technologists make it possible to achieve higher yields and profits

The early-ripening and cold-hardy amaranth cultivar Frant (A. cruentus) was released by the N.I. Vavilov All-Russian Institute of Plant Genetic Resources (VIR) after selecting single-stemmed red-leaved plant forms up to 1.2 m tall from a local population of Indian origin, inbreeding, and free pollination of their linear progeny (Patent, 2022).

## Hybridization

Hybridization is known to be the most widespread and effective breeding technique to obtain new gene combinations. One of the first to classify the interspecies hybridization within the genus Amaranthus was M.J. Murray (1940). He structured amaranth species according to the arrangement of male flowers in the inflorescences, and made a lot of crosses between monoecious and dioecious species. T.N. Khoshoo and M. Pal (1972) during their studies succeeded in hybridizing A. hypochondriacus (served as a pollinator) with A. hybridus and A. caudatus. The F1 hybrids of A. hypochondriacus × A. hybridus had the highest pollen fertility. A weighty contribution to understanding the amaranth gene pool availability was made by E.J. Greizerstein and L. Poggio (1992, 1995) who analyzed the meiotic configuration in 13 different spontaneous amaranth hybrids.

Generally, hybridization is most effective with A. hypochondriacus and A. hybridus, since these species are close in their evolutionary development and contain the same chromosome number (2n = 32). For example, crossing A. hypochondriacus with a Pakistani accession of A. hybridus at the Nebraska Agricultural Experiment Station resulted in the release of a high-yielding cultivar of grain amaranth, Plainsman (PI 358322), widely distributed across the United States. Its characteristics include earliness, high productivity, and the plant height of 1.5–1.8 m (Baltensperger et al., 1992).

Hybridization helped to transfer useful traits from wild amaranth species. For example, the traits of wild A. powellii were implanted into the breeding lines of A. cruentus and A. hypochondriacus to reduce seed shattering. Hybrids with the wild dioecious species A. cannabinus exhibited increased seed size. Resistance of A. hybridus to herbicides was transferred into the breeding lines of A. hypochondriacus and A. cruentus (Brenner et al., 2000).

Interspecies hybridization between grain amaranth species and vegetable ones often results in hybrids with teratological manifestations, high pollen sterility, and chromosomal aberrations, evidencing the existence of a significant incompatibility barrier between them (Mohindeen, Irulappan, 1993). Intraspecies hybridization within A. hypochondriacus failed to reveal any heterotic effect in the progeny. A similar result was observed for crosses among representatives of A. cruentus. Heterosis, however, was registered for interspecies crosses between A. cruentus and A. hypochondriacus, resulting in a statistically significant increase in the progeny’s leaf biomass (Lehmann et al., 1991). M.G. Stetter et al. (2016) developed an effective technique for producing intra- and interspecies amaranth hybrids, which included immersing inflorescences in a water bath at 45 °C for 10 min to emasculate male flowers, as well as the SNP markers for their identification.

Male sterility is reproductive failure in some plants, where the male organs in hermaphrodite flowers are nonfunctional and produce nonviable pollen grains. It is widely used by plant breeders and commercial producers of hybrids. For amaranths, cytoplasmic male sterility (CMS) is a rare phenomenon, identified only in one species, A. hypochondriacus (Peters, Jain 1987; Brenner, 1993). Kenyan breeders (Gudu, Gupta 1988) pinpointed twenty plants with male sterility in a population of cv. Jumla. As a result of a long-term research, David Brenner from Iowa State University, USA, registered the first CMS line of amaranth, DB 199313, and selected a sterility maintainer for it (Brenner, 2019). The first CMS-based amaranth hybrid is likely to be expected in the nearest future.

Diallelic crosses among six genotypes of A. hypochondriacus (F1 and F2 ) were made to analyze protein content in amaranth seeds (Pandey, Pal, 1985). The resulting hybrids exceeded the average parental value in the studied character, and the hybrids from three of those crosses surpassed the best parent. These results confirmed the positive effect of hybridization on the breeding process aimed at higher protein content in seeds, an important trait for amaranth.

Mutation breeding

The diversity of genetic combinations can be increased through both classical hybridization and mutagenesis. The frequency of spontaneous mutations is fairly low, and plant breeders induce them artificially with physical or chemical mutagens. This approach proved its efficiency for crop improvement. Mutation breeding of amaranths for higher seed quality and quantity has mainly been based on the use of radiation mutagenesis. For example, the radiation method (175 Gray) was used to develop mutant lines of A. cruentus, which reliably demonstrated a stable 1,000 seed weight increase in the M4 and M5 generations (Gajdošová et al., 2007). In 2009, mutants were obtained from the local Peruvian cultivar Selection Ancash, with higher concentrations of micronutrients and improved bioavailability due to a decrease in the content of phytic acid (Gómez-Pando et al., 2009). The 2022 studies resulted in the production of six mutant amaranth lines with resistance to soil salinity (Kpochemè et al., 2022). A team of Russian researchers used sodium azide treatment to develop salt-tolerant amaranth forms promising for further breeding; their seeds showed an increase in protein content by 52 % and that of linolenic acid by 25 % (Taipova et al., 2022).

Polyploidy

Polyploidy is regarded as an important evolutionary process for many crop species. Artificially induced polyploidy is the most rapid method to produce new genotypes. It was as early as 50 years ago that a number of plant breeders from various countries started attempting to raise the productivity of grain and vegetable amaranths by inducing polyploidy with colchicine (Behera et al., 1974; Madhusoodanan, Pal, 1984; Sun Y., Yue, 1993). They described some morphological and phenological features in the produced plants: shortening and thickening of the stem, an increase in seed size by 42–159 %, and the onset of flowering occurring one week later than usual. Notably, tetraploids of A. caudatus manifested an increase in the content of protein (by 60 %), amino acids, lysine, and threonine. The results showed that polyploidy in amaranth led to an increase in grain size without a decrease in productivity or nutritional value, confirming the value of this method for breeding programs.

## Genetic engineering and molecular genetics methods

The progress in molecular biology over the past decades has considerably added to the knowledge required for plant genetic diversity management, contributing to the significant advancement of molecular genetics methods in breeding practice. Marker-assisted selection (MAS), being one of such methods, increased the efficiency of selection for a specific trait, and genetic engineering made it possible to transfer a gene from one plant organism to another

Despite the great potential of amaranth, little research has been dedicated to the study of its genomics, concentrating mainly on the identification of its species diversity (Table 2). A genome-wide association study (GWAS) identified associations among specific phenotypes and genomic variants in 10 qualitative traits of amaranth (Jamalluddin et al., 2022). A total of 22 associated markers for inflorescence, leaf, petiole and stem pigmentation were identified on 16 chromosomes in 16 amaranth species. These SNP markers are sources of valuable genetic information for those engaged in phenotyping different amaranth species and improving their cultivars. However, no reports have yet appeared concerning the development of markers for valuable biochemical parameters in amaranth.

**Table 2.**
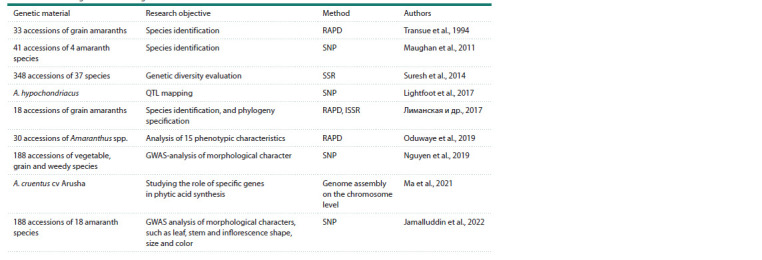
The use of genetic technologies in amaranth studies

Russian researchers (Shcherban, Stasyuk, 2020) undertook to analyze the polymorphism of the gene encoding the squalene synthase (SQS) enzyme in a number of grain and vegetable amaranths. They reported a low level of polymorphism and conservatism of the main functional domains in the gene’s coding part. The data obtained by the authors can help to select grain amaranth forms with higher squalene concentration in seeds

In 1997, a case study of cv. Azteca demonstrated the results of an Agrobacterium-mediated transformation in A. hypochondriacus (Jofre-Garfias et al., 1997). The authors were the first to develop a regeneration technique and an Agrobacterium- mediated system for the crop’s transformation, using them to analyze the expression of the light-harvesting chlorophyll a/b-binding (Lhcb) protein gene promoter in transgenic plants. A team of Indian scientists studied the potential of Agrobacterium-mediated genetic transformation in A. tricolor introducing Ti-plasmid-based constructs with transgenes that enhanced resistance to biotic stresses caused by fungal pathogens, viruses, and pests (Pal et al., 2013). Their efforts resulted in the creation of a reproducible genetic transformation protocol that could be used to produce amaranth plants resistant to biotic factors. U. Munusamy et al. (2013) pioneered in reporting a successful Agrobacterium-mediated transformation of flowers in the inflorescence of A. hypochondriacus. This accomplishment expanded the possibilities of amaranth improvement, as it is not always possible to achieve differentiation of the shoots from a transformed hypocotyl callus (Murugan, Sathishkumar, 2016). For A. cruentus, a successful Agrobacteriummediated transformation from epicotyl explants was reported by Russian authors (Taipova et al., 2020), with the efficiency percentage of 4 %.

Genome-editing tools can also serve to enhance gene efficiency in other crops. For example, protein-coding genes of amaranth have been used to produce transgenic plants of potato, bread wheat, and maize. A 1992 publication (Raina, Datta, 1992) reported successful molecular cloning of AmA1, a protein-coding gene from amaranth seeds with a balanced amino acid composition. Later, a team of Indian scientists succeeded in incorporating this gene into potato, which increased the total protein content in tubers by 60 % (Chakraborty et al., 2000). It is noteworthy that the transgenic potato manifested enhanced photosynthetic activity and higher leaf biomass, with an additional positive effect on the overall yield (Chakraborty et al., 2010).

The same gene of amaranth was used to transform bread wheat, increasing its content of essential amino acids, since bread wheat is known to have a severe deficiency in lysine, threonine, and tyrosine (Tamás et al., 2009). Scientists from the Mexican Center for Research and Advanced Studies (Centro de Investigación y de Estudios Avanzados) used the 11S globulin DNA from A. hypochondriacus to transform the genotype of tropical maize, producing transgenic maize plants with a superexpressed 11S globulin gene which encoded one of the storage proteins in amaranth seeds. As a result, the total protein content in maize seeds showed a 32 % increase (Rascon-Cruz et al., 2004).

Advances in genetic transformation will make it possible to enhance various traits of grain amaranth through genome editing in the nearest future.

## Potential of VIR’s amaranth collection for breeding

As of 2023, 35 amaranth cultivars were listed in the State Register of the Russian Federation (State Register for Selec- tion Achievements Admitted for Usage, 2023). The “oldest” among those, cv. Cherginsky, dates back to 1995; it represents the most numerous group of cultivars earmarked for animal feed purposes (17 in total). The grain group consists of three cultivars, the ornamental group of 10, and the vegetable one of 5. It is worth mentioning that domestic breeding achievements for the amaranth crop are insufficient both in quantity and in the diversity of uses.

**Fig. 2.**
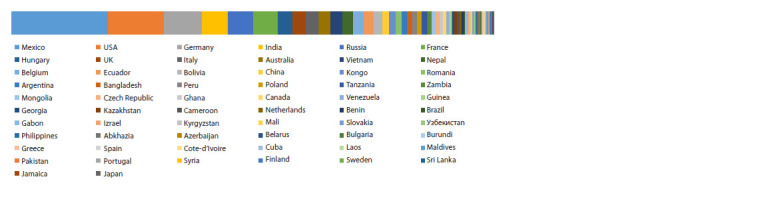
Origin of amaranth accessions maintained at VIR.

The unique collection of amaranth maintained at VIR, currently holding 570 accessions from various countries, is unmatched in the world (Fig. 2).

**Fig. 3.**
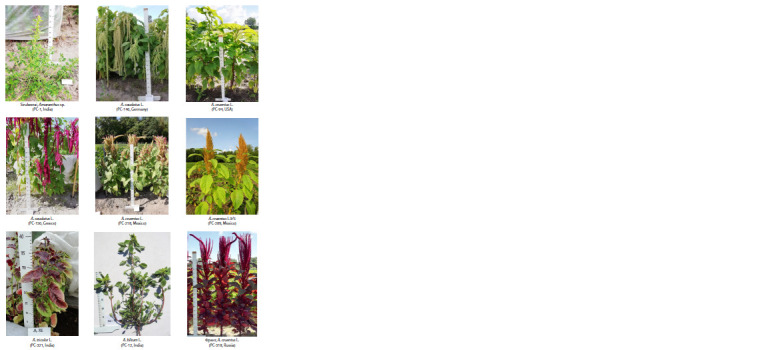
Amaranth accessions maintained at VIR (the photos were taken in the fields of Pushkin and Pavlovsk Laboratories of VIR, St. Petersburg).

The first accession, Sirukeerai (Amaranthus sp., PC-1), arrived from Bangalore Nursery and Gardens, India, in 1955. The collection was subsequently supplied with local cultivars and wild species by numerous collecting missions and shipments from various research centers, botanical gardens, genebanks, and breeding stations all over the world. The largest numbers of accessions came from Mexico, the USA, Germany, and India (Fig. 3)

**Fig. 4.**
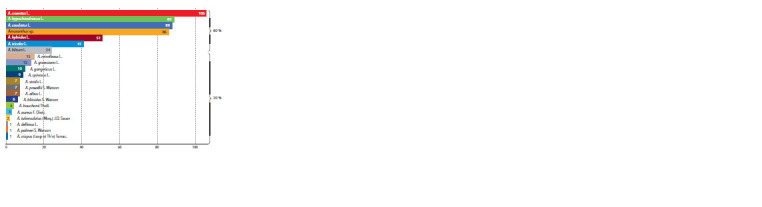
Amaranth species diversity maintained at VIR.

A. cruentus accounts for 80 % of the amaranth collection (106 accessions), followed by A. hypochondriacus (89 accessions), A. caudatus (88), Amaranthus sp. (86), A. hybridus (51), and A. tricolor (41) (Fig. 4). Most of the species in the collection are monoecious. Accessions of A. tuberculatus and A. palmeri are dioecious

The amaranth germplasm collection preserved at VIR undergoes comprehensive studies: useful agronomic traits of the accessions are evaluated, including biochemical indica- tors, morphological descriptions are produced, and utilization areas are specified. The identified valuable biotypes are grouped into trait-specific collections. On the basis of the data procured during long-term research, the amaranth accessions have been distributed into groups according to their best features: high seed yield, high leaf biomass, increased protein content in seeds, short stem, earliness, cold hardiness, resistance to seed shattering, and fitness for vegetable or ornamental use.

VIR has been conducting a study of the amino acid composition in the leaf biomass of cultivated vegetable and grain amaranths as well as wild species. A case study of accessions belonging to 12 different amaranth species identified 18 free amino acids, with eight of them essential (Sokolova et al., 2021). A number of grain amaranth accessions representing A. caudatus, A. cruentus and A. hypochondriacus were recognized as promising sources of highly balanced amino acid composition in green biomass. It was found within the same study that weedy amaranth species had significant potential in terms of their therapeutic effects on the human organism due to high phenolic and lysine contents in their leaves. An accession of A. blitum (syn. A. lividus) (PC-31, India) was identified as the best for its content of ascorbic acid (90.2 mg/100 g), saccharides, organic acids, phenolic compounds, and fatty acids, as well as for its capability to accumulate up to 90.53 mg/100 g of lysine, and significant amounts of tyrosine, tryptophan, and cystine.

One of the problems with amaranth cultivation in Russia is the crop’s thermophilic nature. The optimum tempera- ture range for seed germination is 20–25 °C (Kononkov, Sergeeva, 2011). That is why amaranth can be grown mainly in the southern regions of Russia. Hence, there is a need to develop cold-hardy cultivars. For many years the amaranth collection of VIR has been assessed for cold hardiness under the conditions of Northwest Russia. Genotypes have been selected for their tolerance to low temperatures, and ability to produce mature seeds within a shorter period. The result of these efforts is cv. Frant released by VIR, with its ability to form seeds in 90 days and provide three cuttings of green biomass for tea production in the environments of Leningrad Province.

## Conclusion

Amaranth is rapidly becoming more and more popular in Russia. Of late, it has received a lot of attention from researchers, medical experts, and crop producers due to its diverse uses, unique biochemical composition, and therapeutic potential. A wide range of genetic variability demonstrated by local cultivars of some amaranth species opens up great prospects for its improvement by both conventional and advanced breeding methods

The amaranth collection maintained at VIR, with its nearly seventy-year history, is unique in its origin and diversity. It harbors trait-specific groups of accessions useful for all prioritized breeding trends. The crop’s genetic diversity is highly promising for breeding practice and intensive research in the light of modern knowledge and technologies. Longterm comprehensive studies made it possible to identify amaranth accessions that may be recommended for inclusion in breeding programs. It should be highlighted that the amaranth gene pool preserved at VIR is capable of providing unlimited opportunities for breeding and meeting the needs of the country’s population, enriching the diet with healthfriendly and wholesome products.

## Conflict of interest

The authors declare no conflict of interest.

## References

Abbasi D., Rouzbehan Y., Rezaei J. Effect of harvest date and nitrogen
fertilization rate on the nutritive value of amaranth forage (Amaranthus
hypochondriacus). Anim. Feed Sci. Technol. Animal. 2012;171:
6-13. DOI 10.1016/j.anifeedsci.2011.09.014

Akin-Idowu P., Gbadegesin M., Orkpeh U., Ibitoye D., Odunola O.
Characterization of grain amaranth (Amaranthus spp.) germplasm
in south west Nigeria using morphological, nutritional, and random
amplified polymorphic DNA (RAPD) analysis. Resources. 2016;
5(1):6. DOI 10.3390/resources5010006

Arreguez G.A., Martínez J.G., Ponessa G. Amaranthus hybridus L.
ssp. hybridus in an archaeological site from the initial mid-holocene
in the southern argentinian Puna. Quat. Int. 2013;307:81-85. DOI
10.1016/j.quaint.2013.02.035

Ayorinde F.O. Determination of fatty acid composition of Amaranthus
species. J. Am. Oil Chem. Soc. 1989;66:1812-1814

Baltensperger D.D., Weber L.E., Nelson L.A. Registration of ‘Plainsman’
grain amaranths. Crop Sci. 1992;32:1510-1511. DOI 10.2135/
cropsci1992.0011183X003200060047x

Behera B., Tripathy A., Patnaik S.N. Histological analysis of colchicines-
induced deformities and cytochimeras in Amaranthus caudatus
and A. dubius. J. Heridity. 1974;65:179-184

Brenner D.M. Hybrid seeds for increased amaranth grain yield. Legacy.
1993;6:9-11

Brenner D.M. Registration of DB 199313, cytoplasmic male sterile
grain amaranth genetic stock. J. Plant Regist. 2019;13:251-253. DOI
10.3198/jpr2018.06.0042crgs

Brenner D.M., Baltensperger D.D., Kulakow P.A., Lehmann J.W.,
Myers
R.L., Slabbert M.M., Sleugh B.B. Genetic resources and
breeding in Amaranthus. In: Janick J. (Ed.) Plant Breeding Re-views.
Wiley, New York, 2000;19;227-285. DOI 10.1002/97804706
50172.ch7

Bressani R. Composition and nutritional properties of amaranth. In: Paredes-
Lopez O. (Ed.). Amaranth, Biology, Chemistry and Technology.
Chap. 10. Boca Raton: CRC Press, 1994. DOI 10.1201/9781
351069601-10

Chagaray A. Estudio de factibilidad del cultivo del amaranto. Dirección
Provincial de programación del desarrollo Ministerio de producción
y desarrollo Gobierno de la provincia de Catamarca. Catamarca, Argentina,
2005

Chakraborty S., Chakraborty N., Datta A. Increased nutritive value of
transgenic potato by expressing a nonallergenic seed albumin gene
from Amaranthus hypochondriacus. Proc. Natl. Acad. Sci. USA.
2000;97:3724-3729. DOI 10.1073/pnas.050012697

Chakraborty S., Chakraborty N., Agrawal L., Ghosh S., Narula K.,
Shekhar S., Naikb P.S., Pandec P.C., Chakrborti S.K., Datta A.
Next-generation protein-rich potato expressing the seed protein
gene AmA1 is a result of proteome rebalancing in transgenic tuber.
Proc. Natl. Acad. Sci. USA. 2010;107:17533-17538. DOI 10.1073/
pnas.1006265107

Cheeke P.R., Carlsson R., Kohler G.O. Nutritive value of leaf protein
concentrates prepared from Amaranthus species. Can. J. Anim. Sci.
1981;61:199-204. DOI 10.4141/cjas81-026

Cheng A., Mayes S., Dalle G., Demissew S., Massawe F. Diversifying
crops for food and nutrition security – a case of teff. Biol. Rev.
2015;92:188-198. DOI 10.1111/brv.12225

Costea M., Sanders A., Waines G. Preliminary results towards revision
of the Amaranthus hybridus species complex (Amaranthaceae).
Sida. 2001;19:931-974

Covas G. Perspectivas del cultivo de los amarantos en la republica Argentina.
1993. https://www.semanticscholar.org/paper/Perspectivasdel-
cultivo-de-los-amarantos-en-la-Covas/c2d32274738dbf8eb28
950d2f077f6809c3b132f

Das S. Taxonomical observation on the grain amaranths and new varieties
of Amaranthus cruentus (Amaranthaceae). Nord. J. Bot. 2012;
30:412-420. DOI 10.1111/j.1756-1051.2011.01383.x

Das S. Amaranthus: A Promising Crop of FUTURE. Springer, Singapore,
2016. DOI 10.1007/978-981-10-1469-7

Dawson I.K., Park S.E., Attwood S.J., Jamnadass R., Powell W., Sunderland
T., Carsan S. Contributions of biodiversity to the sustainable
intensification of food production. Global Food Secur. 2019;21:23-
37. DOI 10.1016/j.gfs.2019.07.002

De Montellano B.R.O. Aztec medicine, health, and nutrition. Rutgers
University Press, Great Britain, 1990

Ebert A. Potential of underutilized traditional vegetables and legume
crops to contribute to food and nutritional security, income and more
sustainable production systems. Sustainability. 2014;6:319-335.
DOI 10.3390/su6010319

Espitia E. Amaranth germplasm development and agronomic studies
in Mexico. Food Rev. Int. 1992;8:71-86. DOI 10.1080/875591292
09540930

Evon P., de Langalerie G., Labonne L., Merah O., Talou T., Ballas S., Véronèse
T. Low-density insulation blocks and hardboards from ama-ranth
(Amaranthus cruentus) Stems, a new perspective for building
applications. Coatings. 2021;11:349. DOI 10.3390/coatings11030349

Gajdošová A., Libiaková G., Fejér J. Improvement of selected Amaranthus
cultivars by means of mutation induction and biotechnological
approaches. 2007. In: Ochatt S., Mohan Jain S. (Eds.). Breeding of
Neglected and Under-utilized Crops, Spices and Herbs. Edenbridge
Ltd. USA, 2007;151-169

Gamel T.H., Mesallam A.S., Damir A.A., Shekib L.A., Linssen J.P.
Characterization of amaranth seed oils. J. Food Lipids. 2007;14:
323-334

Gómez-Pando L.R., Eguiluz A., Jiménez J., Falconi J., Aguilar E.,
Shu Q. Barley (Hordeun vulgare) and kiwicha (Amaranthus caudatus)
improvement by mutation induction in Peru. In: Shu Q.Y. (Ed.).
Induced Plant Mutation in the Genomics Era. Food and Agriculture
Organization of the United Nations, Rome, 2009;330-332

Gonor K.V., Pogozheva A.V., Derbeneva S.A., Maltsev G.Yu., Trushina
E.N., Mustaphina O.K. The influence of a diet with including amaranth
oil antioxidant and immune status in patients with ischemic
heart disease and hyperlipoproteidemia. Voprosy Pitaniya = Problems
of Nutrition. 2006;75(6):30-33. (in Russian)

Greizerstein E.J., Poggio L. Estudios citogeneticos de seis hibridos interespecificos
de Amaranthus (Amaranthaseae). Darwiniana. 1992;
31:159-165

Greizerstein E.J., Poggio L. Meiotic studies of spontaneous hybrids of
Amaranthus: genome analysis. Plant Breed. 1995;114:448-450

Grobelnik-Mlakar S., Turinek M., Jakop M., Bavec M., Bavec F. Nutrition
value and use of grain amaranth: potential future application in
bread making. Agricultura. 2009;6:43-53

Gudu S., Gupta V.K. Male-sterility in the grain amaranth (Amaranthus
hypochondriacus ex-Nepal) variety Jumla. Euphytica. 1988;37:23-
26. DOI 10.1007/BF00037218

Hauptli H., Jain S.K. Biosystematics and agronomic potential of some
weedy and cultivated amaranths. Theor. Appl. Genet. 1978;52:177-
185. DOI 10.1007/bf00282575

Hauptli H., Jain S. Genetic variation in outcrossing rate and correlated
floral traits in a population of grain amaranth (Amaranthus cruentus
L.). Genetica. 1985;66:21-27. DOI 10.1007/bf00123602

He H.P., Corke H. Oil and squalene in Amaranthus grain and leaf.
J. Agric. Food Chem. 2003;51:7913-7920. DOI 10.1021/jf030489q

Huang Z.R., Lin Y.K., Fang J.Y. Biological and pharmacological activities
of squalene and related compounds: potential uses in cosmetic
dermatology. Molecules. 2009;14:540-554. DOI 10.3390/
molecules14010540

Jamalluddin N., Massawe F.J., Mayes S., Ho W.K., Symonds R.C.
Genetic diversity analysis and marker-trait associations in Amaranthus
species. PLoS One. 2022;17:0267752. DOI 10.1371/journal.
pone.0267752

Jofre-Garfias A.E., Villegas-Sepúlveda N., Cabrera-Ponce J.L., Adame-
Alvarez R.M., Herrera-Estrella L., Simpson J. Agrobacterium-mediated
transformation of Amaranthus hypochondriacus: light- and
tissue-specific expression of a pea chlorophyll a/b-binding protein
promoter. Plant Cell Rep. 1997;16:847-852. DOI 10.1007/
s002990050332

Joshi B.D., Rana R.S. Grain Amaranths: the Future Food Crops. New
Delhi: National Bureau of Plant Genetic Resources, 1991

Joshi B.D., Mehra K.L., Sharma S.D. Cultivation of grain amaranth in
the north-western hills. Indian Farming. 1983;32:34-37

Joshi D.C., Sood S., Hosahatti R., Kant L., Pattanayak A., Kumar A.,
Yadav D., Stetter M.G. From zero to hero: the past, present and
future of grain amaranth breeding. Theor. Appl. Genet. 2018;131:
1807-1823. DOI 10.1007/s00122-018-3138-y

Kauffman C.S. Thoughts on the development of improved varieties of
grain amaranth. In: Proceedings Third Amaranth Conference, Grain
Amaranth: Expanding Consumption through Improved Cropping,
Marketing and Crop Development. Rodale Press, USA, 1984

Kauffman C.S. Realizing the potential of grain amaranth. Food Rev. Int.
1992;8:5-21. DOI 10.1080/87559129209540927

Kauffman C.S., Weber L.E. Grain amaranth. In: Advances in New
Crops. Portland: Timber Press, 1990;127-139

Khoshoo T.N., Pal M. Cytogenetic pattern in Amaranthus. Chromosomes
Today. 1972;3:259-267

Khoury C.K., Bjorkman A.D., Dempewolf H., Ramirez-Villegas J.,
Guarino L., Jarvis A., Rieseberg L.H., Struik P.C. Increasing homogeneity
in global food supplies and the implications for food security.
Proc. Natl. Acad. Sci. USA. 2014;111:4001-4006. DOI 10.1073/
pnas.1313490111

Kononkov P.F., Gins V.K., Gins M.S. Amaranth is a Promising Crop of
the 21st Century. Moscow, RUDN University Publ., 1999 (in Russian)

Kononkov P.F., Sergeeva V.A. Amaranth – the valuable vegetable and
forage crops multifaceted use. Agrarnyy Vestnik Urala = Agrarian
Bulletin of the Urals. 2011;4:63-64 (in Russian)

Kpochemè A.O.E.K., Hotegni N.F., Missihoun A.A., Gnanvi B.N.,
Atou R., Wouyou A., Montcho D., Gandonou C.B., Agbangla C.,
Ahoton L. Morphological characterization of Amaranthus cruentus
L. mutant lines derived from local and preferred Amaranthus
cultivar. Int. J. Biol. Chem. Sci. 2022;16:1554-1569. DOI 10.4314/
ijbcs.v16i4.16

Lehmann J.W., Clark R.L., Frey K.J. Biomass heterosis and combining
ability in interspecific and intraspecific matings of grain amaranths.
Crop Sci. 1991;31:1111-1116. DOI 10.2135/cropsci1991.0011183x
003100050004x

Leon-Camacho M., Garcia-Gonzalez D.L., Aparicio R. A detailed and
comprehensive study of amaranth (Amaranthus cruentus L.) oil fatty
profile. Eur. Food Res. Technol. 2001;213:349-355. DOI 10.1007/
s002170100340

Lightfoot D.J., Jarvis D.E., Ramaraj T., Lee R., Jellen E.N., Maughan
P.J. Single-molecule sequencing and Hi-C-based proximityguided
assembly of amaranth (Amaranthus hypochondriacus) chromosomes
provide insights into genome evolution. BMC Biology.
2017;15:74. DOI 10.1186/s12915-017-0412-4

Lymanska S.V., Miroshnichenko L.A., Goptsiy T.I., Korneeva O.S.
Polymorphism of RAPD and ISSR markers in grain amaranth species. Vavilovskii Zhurnal Genetiki i Selektsii = Vavilov Journal
of Genetics and Breeding. 2017;21(2):189-197. DOI 10.18699/
VJ17.236

Ma X., Vaistij F.E., Li Y., Van Rensburg W.S.J., Harvey S., Bairu M.W.,
Venter S.L., Mavengahama S., Ning Z., Graham I.A., Deynze A.V.,
Peer Y.V., Denby K.J. A chromosome-level Amaranthus cruentus
genome assembly highlights gene family evolution and biosynthetic
gene clusters that may underpin the nutritional value of this traditional
crop. Plant J. 2021;107:613-628. DOI 10.1111/tpj.15298

Madhusoodanan K.J., Pal M. Autotetraploids in Amaranthus tricolor
Linn. Indian J. Genet. 1984;44:181-185

Magomedov I.M., Chirkova T.V. Amaranth – past, present and future.
Uspekhi Sovremennogo Yestestvoznaniya = Advances in Сurrent
Natural Sciences. 2015;1:1108-1113 (in Russian)

Magomedov I.M., Chirkova А.I., Chirkova T.V. The role of biopeptides
and antioxidants from amaranth grain in the prevention of chronic
human diseases. Prakticheskaya Fitoterapiya = Practical Phytotherapy.
2017;2:49-54 (in Russian)

Mangelsdorf P.C. Genetic potentials for increasing yields of food crops
and animals. Proc. Natl. Acad. Sci. USA. 1966;56:370-375. DOI
10.1073/pnas.56.2.370

Maughan P., Smith S., Fairbanks D., Jellen E. Development, characterization,
and linkage mapping of single nucleotide polymorphisms
in the grain amaranths (Amaranthus sp.). Plant Gen. 2011;4:92. DOI
10.3835/plantgenome2010.12.0027

Mayes S., Massawe F.J., Alderson P.G., Roberts J.A., Azam-Ali S.N.,
Hermann M. The potential for underutilized crops to improve security
of food production. J. Exp. Bot. 2011;63:1075-1079. DOI
10.1093/jxb/err396

Miettinen T.A., Vanhanen H. Serum concentration and metabolism of
cholesterol during rapeseed oil and squalene feeding. Am. J. Clin.
Nutr. 1994;59(2):356-363. DOI 10.1093/ajcn/59.2.356

Mohindeen H.K., Irulappan I. Improvement in amaranths. In: Chadha
K.L., Kalloo G. (Eds.) Advances in Horticulture: Vegetable
Crops. New Delhi: Malhotra Publishing House, 1993

Munusamy U., Abdullah S.N.A., Aziz M.A., Khazaai H. Female reproductive
system of Amaranthus as the target for Agrobacteriummediated
transformation. Adv. Biosci. Biotechnol. 2013;4:188-192.
DOI 10.4236/abb.2013.42027

Murray M.J. The genetics of sex determination in the family Amaranthaceae.
Genetics. 1940;25:409-431. DOI 10.1093/genetics/25.4.409

Murugan S.B., Sathishkumar R. Establishment of high frequency callus
induction and genetic transformation in neglected leafy vegetable
Amaranthus trisis. Austin J. Biotechnol. Bioeng. 2016;3:1058

Myers R.L., Putnam D.H. Growing grain amaranth as a specialty crop.
Center for alternative crops and products, university of Minnesota.
1988. https://conservancy.umn.edu/items/d6b7c0a1-6c3e-45a8-bf8d-
08ab79a5cdb6 (date of access: 28.06.2024)

Nguyen D.C., Tran D.S., Tran T.T.H., Ohsawa R., Yoshioka Y. Genetic
diversity of leafy amaranth (Amaranthus tricolor L.) resources in
Vietnam. Breed. Sci. 2019;69:640-650. DOI 10.1270/jsbbs.19050

Oduwaye O.A., Ayo-Vaughan M.A., Porbeni J.B.O., Oyelakin O.O.
Genetic diversity in Amaranth (Amaranthus spp.) based on phenotypic
and RAPD markers. Nigerian J. Biotechnol. 2019;36:62-68.
DOI 10.4314/njb.v36i1.9

Pal A., Swain S.S., Das A.B., Mukherjee A.K., Chand P.K. Stable germ
line transformation of a leafy vegetable crop amaranth (Amaranthus
tricolor L.) mediated by Agrobacterium tumefaciens. In Vitro Cell.
Dev. Biol.-Plant. 2013;49:114-128. DOI 10.1007/s11627-013-9489-9

Palombini S.V., Claus T., Maruyama S.A., Gohara A.K., Souza A.H.P.,
Souza N.E., Visentainer J.V., Gomes S.T.M., Matsushita M. Evaluation
of nutritional compounds in new amaranth and quinoa cultivars.
Food Sci. Technol. 2013;33:339-344. DOI 10.1590/s0101-
20612013005000051

Pandey R.M., Pal M. Genetics of grain protein in Amaranthus. Crop
Improv. 1985;12:55-58

Patent, 2022. https://gossortrf.ru/registry/gosudarstvennyy-reestr-selek
tsionnykh-dostizheniy-dopushchennykh-k-ispolzovaniyu-tom-
1-sorta-rasteni/frant-amarant-metelchatyy/ (date of access:
04.11.2024) (in Russian)

Peters I., Jain S. Genetics of grain amaranths. Gene-cytoplasmic malesterility.
J. Heredity. 1987;78:251-256. DOI 10.1093/oxfordjournals.
jhered.a110377

Raiger H.L., Bhandari D.C. Underutilized crops: varieties released in
India. All India Coordinated Research Network on Underutilized
Crops. New Daihi: NBPGR, 2012

Raina A., Datta A. Molecular cloning of a gene encoding a seed-specific
protein with nutritionally balanced amino acid composition from
Amaranthus. Proc. Natl. Acad. Sci. USA. 1992;89:11774-11778.
DOI 10.1073/pnas.89.24.11774

Ramdwar M.N., Chadee S.T., Stoute V.A. Estimating the potential consumption
level of amaranth for food security initiatives in Trinidad,
West Indies. Cogent Food Agric. 2017;3:1321475. DOI 10.1080/
23311932.2017.1321475

Rao C.V., Newmark H.L., Reddy B.S. Chemopreventive effect of
squalene on colon cancer. Carcinogenesis. 1998;19:287-290. DOI
10.1093/carcin/19.2.287

Rascón-Cruz Q., Sinagawa-García S., Osuna-Castro J.A., Bohorova N.,
Paredes-López O. Accumulation, assembly, and digestibility of amarantin
expressed in transgenic tropical maize. Theor. Appl. Genet.
2004;108:335-342. DOI 10.1007/s00122-003-1430-x

Rezaei J., Rouzbehan Y., Fazaeli H. An assessment of digestibility and
protein quality of the fresh and ensiled amaranth forage according to
CNCPS. Iranian J. Anim. Sci. 2009;40:31-38

Ruth O.N., Unathi K., Nomali N., Chinsamy M. Underutilization
versus nutritional-nutraceutical potential of the Amaranthus food
plant: a mini-review. Appl. Sci. 2021;11:6879. DOI 10.3390/
app11156879

Sauer J.D. The grain amaranths and their relatives: a revised taxonomic
and geographic survey. Ann. Mo. Bot. Gard. 1967;54:103-137. DOI
10.2307/2394998

Saunders R.M., Becker R. Amaranthus: a potential food and feed resource.
In: Pomeranz Y. (Ed.). Advances in Cereal Science and Technology.
St. Paul: American Association of Cereal Chemists, 1984;
357-396

Schulz-Schaeffer J., Stallknecht G.F., Baldridge D.E., Larson R.A.
Registration of Montana-3 grain amaranth germplasm. Crop Sci.
1989a;29:244-245

Schulz-Schaeffer J., Webb D.M., Baldridge D.E., Stallknecht G.F.,
Larson R.A. Registration of Montana-5 grain amaranth germplasm.
Crop Sci. 1989b;29:1581

Schulz-Schaeffer J., Baldridge D.E., Bowman H.F., Stallknecht G.F.,
Larson R.A. Registration of ‘Amont’ grain amaranth. Crop Sci.
1991;31:482-483

Shadi H., Rouzbehan Y., Rezaei J., Fazaeli H. Yield, chemical composition,
fermentation characteristics, in vitro ruminal variables, and
degradability of ensiled amaranth (Amaranthus hypochondriacus)
cultivars compared with corn (Zea mays) silage. Transl. Anim. Sci.
2020;4:1-12. DOI 10.1093/tas/txaa180

Shcherban A.B., Stasyuk A.I. Polymorphism of the squalene synthase
gene (SQS) in different species of amaranth (Amaranthus L.). Russ.
J. Genet. 2020;56:298-306. DOI 10.31857/S0016675820030145

Singh A., Mahato A.K., Maurya A., Rajkumar S., Singh A.K., Bhardwaj
R., Kaushik S.K., Kumar S., Gupta V., Singh K., Singh R.
Amaranth Genomic Resource Database: an integrated database resource
of Amaranth genes and genomics. Front. Plant Sci. 2023;14:
1203855. DOI 10.3389/fpls.2023.1203855

Sleugh B.B., Moore K.J., Brummer E.C., Knapp A.D., Russell J., Gibson
L. Forage value of various amaranth species at different harvest
dates. Crop Sci. 2021;41:466-472. DOI 10.2135/cropsci2001.
412466x

Smith M.E. The Aztecs. Blackwell, Oxford, 1996

Smith T.J. Squalene: potential chemopreventive agent. Expert Opin.
Invest. Drugs. 2000;9:1841-1848. DOI 10.1517/13543784.9.8.1841

Sokolova D., Zvereva O., Shelenga T., Solovieva A. Comparative characteristics
of the amino acid composition in amaranth accessions
from the VIR collection. Turk. J. Agric. For. 2021;45:68-78. DOI
10.3906/tar-2007-7

Soriano-Garcıa M., Arias-Olguín I.I., Montes J.P.C., Ramırez D.G.R.,
Silvestre Mendoza Figueroa J., Flores-Valverde E., Rodrguez M.R.V.
Nutritional functional value and therapeutic utilization of amaranth.
J. Analytical Pharm. Res. 2018;7(5):596-600. DOI 10.15406/japlr.
2018.07.00288

Sreelathakumary I., Peter K.V. Amaranth-Amaranthus spp. In: Kallo
G., Bergh B.O. (Eds.). Genetic Improvement of Vegetable Crops.
Oxford: Pergamon Press, 1993; 315-323

Stallknecht G.F., Schulz-Schaefer J.R. Amaranth rediscovered. In:
Janick J., Simon J.E. (Eds.). New Crops. New York: Wiley, 1993

State Register of Selection Achievements Admitted for Usage,
2023. https://gossortrf.ru/publication/reestry.php (date of access:
24.04.2024). (in Russian)

Stetter M.G., Zeitler L., Steinhaus A., Kroener K., Biljecki M.,
Schmid K.J. Crossing methods and cultivation conditions for rapid
production of segregating populations in three grain amaranth species.
Front. Plant Sci. 2016;7:816. DOI 10.3389/fpls.2016.00816

Sun Y., Yue S. Research on polyploid grain amaranth – a preliminary
study on selection of grain amaranth with character of bigger seed
(in Chinese). In: Yue S. (Ed.). The Research and Development of
Grain Amaranth in China. Beijing, China, 1993

Sun G.Q., Ma J., Du W., Wang Y., Li S.L., Xiong Y., Yu X., Lei X.,
Yabin M.L. Effects of dietary supplementation of Amaranthus hypochondriacus
L. on Ruminal fermentation, blood indicators and performance
of dairy cows. Chin. J. Animal Nutr. 2017;29:1652-1660

Suresh S., Chung J.-W., Cho G.-T., Sung J.-S., Park J.-H., Gwag J.-G.,
Baek H.-J. Analysis of molecular genetic diversity and population
structure in Amaranthus germplasm using SSR markers. Plant Biosyst.
– Int. J. Dealing Aspects Plant Biosyst. 2014;148:635-644. DOI
10.1080/11263504.2013.788095

Taipova R.M., Musin K.G., Kuluev B.R. Agrobacterium-mediated transformation
of Amaranthus cruentus L. epicotils. Zhurnal Sibirskogo
Federal’nogo Universiteta. Seriya: Biologiya = Journal of Siberian
Federal University Biology. 2020;13:179-187. DOI 10.17516/1997-
1389-0292 (in Russian)

Taipova R.M., Nesterov V.N., Rozentsvet O.A., Kuluev B.R. Changes
in the content of proteins and lipids and in the state of the antioxidant
system in mutant forms of Amaranthus cruentus L. Trudy po Prikladnoy
Botanike, Genetike i Selektsii = Proceedings on Applied Botany,
Genetics and Breeding. 2022;183:76-85. DOI 10.30901/2227-8834-
2022-1-76-85 (in Russian)

Tamás C., Kisgyörgy B.N., Rakszegi M., Wilkinson M.D., Yang M.- S.,
Láng L., Tamas L., Bedő Z. Transgenic approach to improve wheat
(Triticum aestivum L.) nutritional quality. Plant Cell Rep. 2009;28:
1085-1094. DOI 10.1007/s00299-009-0716-0

Tang Y., Tsao R. Phytochemicals in quinoa and amaranth grains and
their antioxidant, anti‐inflammatory, and potential health beneficial
effects: A review. Mol. Nutr. Food Res. 2017;61:1600767. DOI
10.1002/mnfr. 201600767

Teutonico R.A., Knorr D. Amaranth: composition, properties and applications
of a rediscovered food crop. Food Technol. 1985;1:
49-60

Transue D.K., Fairbanks D.J., Robison L.R., Andersen W.R. Species
identification by RAPD analysis of grain amaranth genetic resources.
Crop Sci. 1994;34:1385. DOI 10.2135/cropsci1994.00111
83x003400050044x

Venskutonis P.R., Kraujalis P. Nutritional components of amaranth
seeds and vegetables: A review on composition, properties, and
uses. Compr. Rev. Food Sci. Food Saf. 2013;12:381-412. DOI
10.1111/1541-4337.12021

